# MRI Markers of EDSS ≥ 3 in Relapsing–Remitting Multiple Sclerosis: An Assessment of Lesion Burden, Brain Volumetry, and IVIM-DWI Metrics

**DOI:** 10.3390/brainsci16070743

**Published:** 2026-07-14

**Authors:** Sami A. Alghamdi, Othman I. Alomair, Manal H. Alosaimi, Abdullah H. Abujamea, Salman Aljarallah, Nuha M. Alkhawajah, Yazeed I. Alashban

**Affiliations:** 1 Radiological Sciences Department, College of Applied Medical Sciences, King Saud University, P.O. Box 145111, Riyadh 4545, Saudi Arabia; salghamdi1@ksu.edu.sa (S.A.A.); manalosaimi@ksu.edu.sa (M.H.A.); yalashban@ksu.edu.sa (Y.I.A.); 2 Medical Physics Section, King Saud University Medical City, King Saud University, Riyadh 2925, Saudi Arabia; abujamea@ksu.edu.sa; 3 Department of Medicine, College of Medicine, King Saud University, P.O. Box 145111, Riyadh 2925, Saudi Arabia; saljarallah@ksu.edu.sa (S.A.); nalkhawajah@ksu.edu.sa (N.M.A.)

**Keywords:** relapsing–remitting multiple sclerosis, EDSS, IVIM-DWI, brain volumetry, ROC analysis

## Abstract

**Highlights:**

**What are the main findings?**

- Fractional volumetric measures, particularly BPF and CSF fraction, showed stronger discriminatory performance than absolute brain volume measures for EDSS ≥ 3 stratification in RRMS.
- The combined lesion count + BPF + f model achieved the highest cross-validated discriminatory performance among the evaluated multimodal MRI models, although the incremental improvement over lesion count + BPF was modest.

**What are the implications of the main findings?**

- Multimodal MRI parameters supported stratification between EDSS < 3 and EDSS ≥ 3 in patients with RRMS.
- Further validation in larger longitudinal cohorts incorporating additional clinical and imaging data is warranted.

**Abstract:**

**Background/Objectives:** Disability in relapsing–remitting multiple sclerosis (RRMS) reflects multiple pathological processes that may not be fully captured by individual MRI markers. Because an EDSS score ≥ 3 represents a clinically meaningful threshold of moderate-to-higher disability, this study evaluated the cross-sectional ability of brain volumetric measures and IVIM-DWI parameters to identify patients with RRMS who had EDSS ≥ 3. **Methods:** This retrospective cross-sectional study included 189 patients with RRMS who had complete EDSS, lesion count, IVIM-DWI, and brain volumetric MRI data. Patients were stratified into EDSS < 3 and EDSS ≥ 3 groups. Single-marker ROC analyses were performed for lesion count, IVIM-DWI parameters, absolute volumetric measures, and fractional volumetric measures. Combined ROC models were constructed to assess the discriminatory performance of integrating lesion burden, volumetric MRI, and IVIM-DWI metrics. Internal model stability was evaluated using 5-fold cross-validation. **Results:** Patients with EDSS ≥ 3 had higher lesion count, higher ADC and D values, lower gray matter, white matter, and brain parenchymal volumes, higher CSF volume, higher CSF fraction, and lower brain parenchymal fraction (BPF). In single-marker ROC analysis, BPF and CSF fraction showed the strongest discriminatory performance (AUC = 0.705), followed by lesion count (AUC = 0.696), whereas absolute BV showed minimal discriminatory value (AUC = 0.503). The core lesion count + BPF model achieved an AUC of 0.757 and a cross-validated AUC of 0.737. Adding perfusion fraction (f) produced the numerically highest cross-validated performance, with an AUC of 0.769 and a cross-validated AUC of 0.746, although the incremental improvement over lesion count + BPF was modest and not statistically significant. **Conclusions:** Fractional volumetric measures showed stronger discriminatory performance than absolute BV for EDSS ≥ 3 stratification in RRMS. The lesion count + BPF + f model achieved the highest cross-validated performance among the evaluated MRI models; however, overall discrimination was moderate, and the incremental improvement associated with f was not statistically significant. These exploratory findings require evaluation in larger longitudinal cohorts with external validation.

## 1. Introduction

Multiple sclerosis (MS) is a chronic immune-mediated disease of the central nervous system characterized by inflammatory demyelination, axonal injury, gliosis, and progressive neurodegeneration [1,2,3]. Relapsing–remitting multiple sclerosis (RRMS) is the most common clinical disease course and is characterized by clearly defined relapses followed by periods of partial or complete remission, without continuous disability progression between attacks [4,5]. Although many patients with RRMS remain ambulatory during the early and intermediate phases of the disease, clinically meaningful disability may accumulate over time through both focal inflammatory injury and diffuse neurodegenerative tissue loss [3].

Clinical disability in MS is most commonly quantified using the Expanded Disability Status Scale (EDSS), an ordinal scale ranging from 0 to 10, where 0 indicates a normal neurological examination and higher scores reflect increasing neurological impairment and ambulatory disability [6]. Although EDSS has recognized limitations, including its nonlinear structure and strong weighting toward ambulation at higher scores, it remains the most widely used disability outcome in MS clinical studies and therapeutic trials [6,7]. The EDSS 3 threshold has particular clinical relevance because patients at this level remain fully ambulatory but demonstrate moderate disability in one functional system or mild disability across multiple functional systems [6,7,8]. Large natural history studies have suggested that sustained disability around EDSS 3 represents an important milestone in MS progression, with disability accumulation before this stage being more variable and subsequent progression showing a more homogeneous trajectory [9,10,11]. Consistent with this framework, prior clinical and imaging studies have stratified patients with MS around the EDSS 3 threshold to distinguish lower-disability patients from those with moderate-to-higher disability [12,13,14]. Therefore, EDSS < 3 and EDSS ≥ 3 provide a practical and clinically interpretable threshold for distinguishing lower-disability patients from those with moderate-to-higher EDSS-defined disability.

A persistent challenge in MS research is that clinical disability is not fully explained by conventional lesion metrics alone [15,16,17]. This discrepancy, commonly referred to as the clinico-radiological paradox, reflects pathological processes beyond visible focal white matter lesions, including diffuse normal-appearing tissue injury, gray matter pathology, spinal cord involvement, network disruption, and global brain tissue loss [15,18]. Magnetic resonance imaging (MRI) is fundamental for MS diagnosis, disease monitoring, and assessment of treatment response [19,20,21,22]. However, conventional MRI markers, including T2 lesion count, lesion load, gadolinium-enhancing lesions, and T1-hypointense lesions, incompletely capture the diffuse and neurodegenerative components of MS that contribute to irreversible disability [15,18,23]. Accordingly, quantitative MRI markers that reflect brain tissue loss and microstructural damage may provide complementary information beyond visible lesion burden.

Brain atrophy has emerged as an important MRI marker of neurodegeneration in MS. Global and regional brain volume loss has been associated with physical disability, cognitive impairment, disease progression, and long-term clinical outcomes [24,25,26,27]. The Magnetic Resonance Imaging in Multiple Sclerosis (MAGNIMS) consensus recommendations emphasized the relevance of brain and spinal cord atrophy measures for prognosis and treatment monitoring, while also highlighting the need for methodological standardization before routine clinical implementation [26]. Recent systematic reviews further support the association between MRI-derived brain atrophy and disability, although reported effect sizes vary according to disease phenotype, imaging acquisition, segmentation method, follow-up duration, and disability outcome definition [24,27].

A major methodological consideration in volumetric MRI is the distinction between absolute and fractional volumetric measures. Absolute volumes, such as gray matter (GM) volume, white matter (WM) volume, brain parenchymal volume (BPV), and cerebrospinal fluid (CSF) volume, may be influenced by inter-individual differences in head size, age, sex, and brain reserve. In contrast, fractional measures such as brain parenchymal fraction (BPF) and CSF fraction normalize brain tissue or CSF compartments relative to the total segmented compartment, thereby providing proportional indices of preserved brain tissue and CSF space expansion [25,26]. These normalized measures may be particularly relevant for cross-sectional disability stratification because they reflect the balance between parenchymal tissue preservation and CSF enlargement more directly than raw volume measures alone.

Advanced diffusion MRI may also provide complementary information regarding microstructural lesion injury. Intravoxel incoherent motion diffusion-weighted imaging (IVIM-DWI) separates true molecular diffusion from perfusion-related pseudo-diffusion and generates quantitative parameters including apparent diffusion coefficient (ADC), true diffusion coefficient (D), pseudo-diffusion coefficient (D*), and perfusion fraction (f) [28,29,30,31]. Previous work has shown that IVIM-DWI can characterize MS lesion heterogeneity and distinguish enhancing, non-enhancing, and chronic black-hole lesions [32]. Additional studies have demonstrated associations between IVIM-derived metrics and disability, although lesion burden and clinical variables may remain stronger determinants of EDSS in multivariable models [33]. More recently, IVIM-DWI radiomics has been explored for lesion phenotyping and clinical status classification using machine learning approaches [34]. However, the extent to which lesion-averaged IVIM-DWI metrics improve disability stratification when considered alongside lesion count and volumetric atrophy measures remains unclear.

ROC analysis provides a clinically interpretable approach for evaluating whether continuous imaging markers can distinguish patients across predefined clinical thresholds. By summarizing classification ability using AUC and estimating marker cutoffs, ROC analysis is suitable for assessing whether lesion count, volumetric MRI measures, and IVIM-DWI metrics identify RRMS patients with EDSS ≥ 3 [35,36,37].

Although previous studies have examined associations between brain atrophy, lesion burden, diffusion MRI, and disability in MS, most have evaluated these markers separately or within association-based frameworks. Direct ROC-based comparisons integrating focal lesion burden, absolute and fractional brain volumetric measures, and lesion-averaged IVIM-DWI parameters within the same RRMS cohort remain limited. By evaluating these imaging domains within the same cohort, the present study addresses whether complementary structural, volumetric, and diffusion–perfusion MRI markers can distinguish RRMS patients with EDSS ≥ 3. Therefore, this study aimed to assess these markers, individually and in combined MRI models, for identifying EDSS ≥ 3 status.

## 2. Materials and Methods

### 2.1. Study Design and Patient Cohort

This retrospective cross-sectional imaging study included patients with RRMS aged 18–63 years who underwent brain MRI as part of routine clinical care at King Saud University Medical City between 2019 and 2024.

The initial dataset consisted of 197 patients with available IVIM-DWI measurements. Because the primary aim of the present analysis was to integrate IVIM-DWI and volumetric MRI variables within the same ROC-based framework, only patients with complete EDSS, lesion count, IVIM-DWI, and brain volumetric data were included. Accordingly, the final complete-case analytical cohort consisted of 189 patients. The eight excluded patients lacked data required for the integrated analysis: four had missing EDSS documentation and four lacked usable 3D pre-contrast T1-weighted images for volumetric assessment. Patients were classified into two disability groups: EDSS < 3 and EDSS ≥ 3.

The study was approved by the Institutional Review Board of King Saud University Medical City (IRB No. E23-7517; initial approval date: 22 January 2023; renewal of ethical approval: 30 June 2025). All procedures were conducted in accordance with the Declaration of Helsinki. The requirement for patient consent was waived because of the retrospective nature of the study. EDSS and other clinical data were collected routinely for clinical purposes and were not obtained specifically for this study. All analyzed data were retrieved retrospectively from patients’ medical records and imaging files after de-identification.

### 2.2. Clinical, Demographic, and Imaging Variables

Clinical and demographic variables included age, sex, disease duration, disease-modifying therapy (DMT) status, and EDSS score. EDSS scores were retrospectively extracted from neurology clinical records and reports documented at the time of MRI acquisition or from the nearest available clinical visit surrounding the MRI examination. When more than one EDSS score was available, the EDSS score closest to the MRI date was used. Because EDSS assessment was not prospectively standardized for research purposes, variability in EDSS-to-MRI timing may have existed across patients; therefore, EDSS-defined disability status was interpreted as a retrospective cross-sectional clinical measure rather than a longitudinal outcome. DMT status was categorized as treated or untreated. Lesion count was derived from conventional MRI assessment and was included as an imaging marker of focal lesion burden in single-marker and combined ROC analyses.

### 2.3. MRI Acquisition

All MRI examinations were performed using a 1.5-T GE MRI scanner (GE Healthcare, Waukesha, WI, USA) equipped with an 8-channel phased-array head coil. The MRI protocol included conventional sequences for MS lesion assessment, three-dimensional (3D) T1-weighted imaging for volumetric analysis, and IVIM-DWI for quantitative diffusion–perfusion assessment.

The conventional MRI protocol included fluid-attenuated inversion recovery (FLAIR), axial T2-weighted imaging, 3D pre- and post-contrast T1-weighted imaging, and diffusion-weighted imaging. Three-dimensional pre- and post-contrast T1-weighted images were acquired using a Fast Spoiled Gradient Recalled Echo (FSPGR) sequence with TR = 9 ms, TE = 4 ms, and voxel size = 1.8 × 0.5 × 0.5 mm. IVIM-DWI was acquired using single-shot echo-planar imaging with TR = 4508 ms, TE = 77.5 ms, voxel size = 0.8 × 0.8 × 5 mm, and b-values of 0, 30, 50, 70, 100, 200, 500, and 1000 s/mm^2^ in three orthogonal directions. These acquisition parameters were consistent with the institutional imaging framework previously applied to RRMS cohorts [32].

### 2.4. IVIM-DWI Processing

Apparent diffusion coefficient (ADC) and IVIM parametric maps were generated using the OsiriX plug-in IB Diffusion™ software, version 21.12 (Imaging Biometrics, Elm Grove, WI, USA). IVIM-DWI separates true molecular diffusion from perfusion-related pseudo-diffusion using multi-b-value diffusion acquisitions. A segmented IVIM model was used to estimate true diffusion coefficient (D), pseudo-diffusion coefficient (D*), and perfusion fraction (f), following the same processing framework previously applied in MS cohorts from the same institutional dataset [32].

The b-value inflection point was set at 200 s/mm^2^ according to the processing protocol used in IB Diffusion™. In this segmented approach, the higher b-value range was used primarily to estimate the diffusion-dominant component (D), whereas the lower b-value range contributed to estimation of the perfusion-related components, including D* and f. The f value was estimated as the ratio of the perfusion-related signal component to the total signal decay.

### 2.5. Lesion Identification and Patient-Level IVIM Aggregation

MS lesions were identified on conventional MRI, primarily using FLAIR and T2-weighted images. Lesion count was recorded for each patient based on these lesions and was used as an imaging marker of focal lesion burden. Lesion regions of interest (ROIs) were manually delineated by a single experienced neuroradiologist using ITK-SNAP, version 3.8.0, which is widely used for medical image segmentation and ROI-based analysis [38]. Lesion counting and ROI placement followed a standardized visual assessment protocol across all patients to maintain consistency in lesion identification and IVIM-DWI metric extraction.

Lesion count included all visible MS lesions identified on conventional MRI. Eligible lesions for IVIM-DWI extraction were lesions that were sufficiently visible for ROI placement while allowing avoidance of CSF contamination, partial-volume effects, imaging artifacts, and adjacent non-lesional brain tissue. Lesion volume was not measured in the present analysis. ROIs were placed within representative lesion tissue, and ROI placement was reviewed for anatomical plausibility and image-quality adequacy before extraction of IVIM-DWI metrics.

For each patient, IVIM-derived values were extracted from all eligible lesions. When multiple eligible lesions were present, lesion-level measurements were averaged to generate a single patient-level mean value for each IVIM-DWI parameter. These patient-level mean values for ADC, D, D*, and f were used in all group comparisons, single-marker ROC analyses, and combined ROC models.

### 2.6. Brain Volumetric Assessment

Three-dimensional T1-weighted images were processed using the Computational Anatomy Toolbox (CAT12) implemented in Statistical Parametric Mapping software (SPM12; Wellcome Centre for Human Neuroimaging, London, UK) running under MATLAB R2022b (MathWorks, Natick, MA, USA) [39,40]. All images were visually inspected before processing to ensure adequate image quality and correct anatomical orientation. Volumetric outputs were reviewed for segmentation plausibility, and cases with incomplete or unusable volumetric data were excluded from the complete-case analysis.

Automated tissue segmentation was performed using the standard CAT12 pipeline. No explicit lesion filling was performed before CAT12 segmentation. Therefore, volumetric outputs were interpreted with consideration of the potential influence of MS lesion-related signal abnormalities on automated tissue classification, particularly in patients with higher lesion burden.

Gray matter (GM), white matter (WM), and cerebrospinal fluid (CSF) volumes were extracted for each patient. In this study, brain volume (BV) was used operationally as a total segmented-compartment volume, defined as GM + WM + CSF, and was used as the denominator for fractional measures. Brain parenchymal volume (BPV) was calculated as GM + WM.

Fractional measures were calculated to express brain tissue and CSF compartments relative to BV. Brain parenchymal fraction (BPF) was calculated as BPV divided by BV, whereas CSF fraction was calculated as CSF volume divided by BV. No additional post hoc correction for head size was applied beyond the use of fractional measures calculated relative to BV. Absolute measures included BV, GM volume, WM volume, BPV, and CSF volume. Fractional measures included BPF and CSF fraction. These measures were included because brain atrophy and normalized volumetric indices are clinically relevant MRI markers in MS and have been associated with disability, disease progression, and long-term clinical outcomes [24,25,26,27].

### 2.7. Outcome Definition

The primary outcome was EDSS-defined moderate-to-higher disability, defined as EDSS ≥ 3. Patients were categorized into a lower-disability group (EDSS < 3) and a moderate-to-higher-disability group (EDSS ≥ 3), consistent with the clinical relevance of the EDSS 3 threshold and prior EDSS-based stratification approaches in MS studies [6,9,10,11,12,13]. This binary outcome was used for all single-marker and combined ROC analyses. EDSS was dichotomized at this threshold to align with the ROC-based objective of distinguishing patients across a clinically interpretable disability level; however, this approach does not replace continuous or ordinal EDSS analyses.

### 2.8. Statistical Analysis

Statistical analyses were performed using IBM SPSS Statistics version 26.0 (IBM Corp., Armonk, NY, USA), with additional ROC-based model evaluation and 5-fold cross-validation performed using custom statistical scripts. Continuous variables were summarized as mean ± standard deviation (SD), and categorical variables were summarized as frequencies and percentages. Continuous variables were compared between EDSS groups using independent-samples *t*-tests, whereas categorical variables were compared using chi-square tests or Fisher’s exact tests, as appropriate. Effect sizes for continuous group comparisons were reported using Cohen’s d. A two-sided *p*-value < 0.05 was considered statistically significant. As a supplementary sensitivity analysis, Spearman rank correlation was used to evaluate associations between EDSS as an ordinal/continuous measure and the main MRI markers, including lesion count, BPF, CSF fraction, ADC, D, D*, and f.

Single-marker ROC analysis was performed to evaluate the discriminatory ability of lesion count, IVIM-DWI metrics, and volumetric MRI measures for identifying patients with EDSS ≥ 3. For each marker, the area under the curve (AUC), 95% confidence interval (CI), *p*-value, optimal cutoff, sensitivity, specificity, and Youden index were calculated. The *p*-value indicated whether the AUC differed significantly from 0.5. The optimal cutoff was determined using the Youden index, consistent with standard ROC-based threshold selection approaches [36]. All Youden-derived cutoff values were considered exploratory, data-driven thresholds for descriptive ROC-based stratification and were not interpreted as validated or clinically actionable decision thresholds.

Combined ROC models were constructed using logistic regression to assess the discriminatory performance of integrating lesion count with volumetric MRI and IVIM-DWI metrics. Model-estimated probabilities from each logistic regression model were used to generate ROC curves and calculate model-level AUC values. All multivariable ROC models were interpreted as exploratory internally evaluated cross-sectional stratification models and were not intended for diagnosis, prognosis, treatment decision-making, or clinical implementation.

The lesion count + BPF model was evaluated as the core parsimonious imaging model because it combined focal lesion burden with fractional brain tissue loss. To assess the contribution of IVIM-DWI within this multimodal MRI framework, ADC, D, D*, and f were added separately to the lesion count + BPF model. Additional models evaluated lesion count combined with selected volumetric measures. A broader clinical-imaging model including age, sex, DMT status, lesion count, and BPF was also assessed. To further address potential clinical confounding, an additional adjusted clinical-imaging logistic regression model was evaluated among patients with available disease-duration data. This model included age, sex, disease duration, DMT status, lesion count, BPF, and f. The adjusted model focused on f because the lesion count + BPF + f model showed the highest cross-validated performance among the IVIM-augmented imaging models. ADC, D, D*, and f were not entered simultaneously because these IVIM-DWI metrics are related parameters derived from the same acquisition and could increase the risk of overfitting and potential collinearity. Adjusted odds ratios (ORs), 95% confidence intervals (CIs), *p*-values, model-level AUC, and 5-fold cross-validated AUC were reported.

To evaluate the incremental contribution of f, the AUC difference between the lesion count + BPF model and the lesion count + BPF + f model was assessed using paired bootstrap resampling of model-estimated probabilities. The AUC difference, 95% bootstrap CI, and *p*-value were reported.

BPF and CSF fraction were not included simultaneously in the same model because they are mathematically complementary and may introduce collinearity. Internal model stability was assessed using 5-fold cross-validation, and cross-validated AUC values were reported. For combined models, classification performance at the optimal exploratory cutoff was summarized using sensitivity, specificity, accuracy, and Youden index. For 5-fold cross-validation, the cohort was divided into five folds; in each iteration, the model was trained on four folds and tested on the remaining fold.

Cross-validated AUC values were derived from model-estimated probabilities in the held-out fold across the five cross-validation iterations and were interpreted as an estimate of internal model stability rather than external validation. All models were interpreted as MRI-based disability stratification models rather than diagnostic or longitudinal prognostic models.

## 3. Results

**Figure 1:**
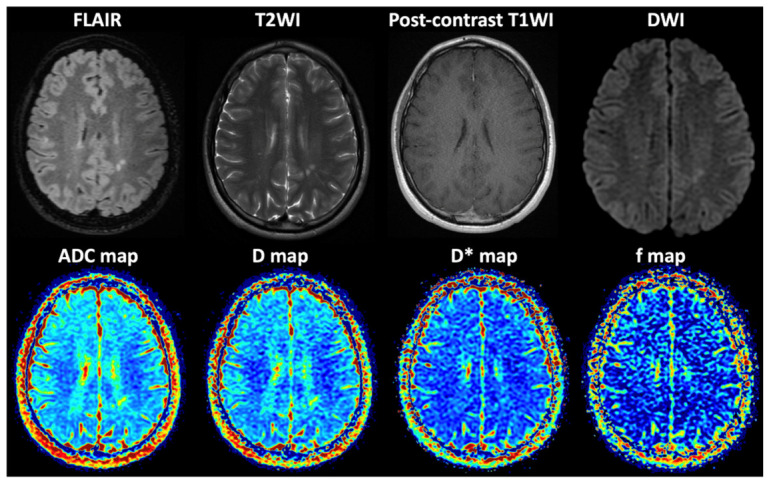
Example multimodal MRI case from the EDSS ≥ 3 group. Images show conventional and quantitative MRI findings in a 32-year-old female patient with RRMS, EDSS score of 7.0, disease duration of 10 years, and 40 MS lesions. The figure includes T2-weighted, FLAIR, post-contrast T1-weighted, DWI, ADC, D, D*, and f-maps. Lesion-averaged IVIM-DWI metrics were ADC = 1.323 × 10^−3^ mm^2^/s, D = 1.260 × 10^−3^ mm^2^/s, D* = 1.513 × 10^−3^ mm^2^/s, and f = 6.19%. Volumetric MRI showed BV = 1376.25 mL, GM volume = 641.59 mL, WM volume = 394.05 mL, BPV = 1035.64 mL, CSF volume = 340.61 mL, BPF = 75.25%, and CSF fraction = 24.75%.

Figure 1 illustrates an example multimodal MRI case from the EDSS ≥ 3 group, demonstrating conventional MRI, IVIM-DWI, and volumetric MRI findings in a patient with high lesion burden and advanced disability.

### 3.1. Study Cohort and Disability Stratification

The final complete-case analytical cohort included 189 patients with RRMS who had complete EDSS, lesion count, IVIM-DWI, and brain volumetric MRI data. Patients were stratified according to disability status into an EDSS < 3 group (*n* = 136) and an EDSS ≥ 3 group (*n* = 53).

Patients with EDSS ≥ 3 were significantly older and had longer disease duration than those with EDSS < 3. The EDSS ≥ 3 group also had a significantly higher lesion count, with a mean of 15.93 ± 9.67 lesions compared with 9.87 ± 6.86 lesions in the EDSS < 3 group (*p* < 0.001; Cohen’s d = 0.78). Among IVIM-DWI metrics, ADC and D were significantly higher in patients with EDSS ≥ 3, whereas D* did not differ significantly between groups. The f value was significantly lower in patients with EDSS ≥ 3, with a small effect size.

Brain volumetric measures also differed between disability groups. Patients with EDSS ≥ 3 had significantly lower GM volume, WM volume, and BPV, together with significantly higher CSF volume, higher CSF fraction, and lower BPF. In contrast, BV did not differ significantly between groups. Detailed group comparisons are presented in Table 1. The full EDSS distribution is provided in Supplementary Table S1. In supplementary sensitivity analysis treating EDSS as an ordinal/continuous measure, the complementary BPF/CSF fraction relationship showed the strongest volumetric association with EDSS (CSF fraction: ρ = 0.411, *p* < 0.001; BPF: ρ = −0.410, *p* < 0.001), followed by lesion count (ρ = 0.368, *p* < 0.001), D (ρ = 0.339, *p* < 0.001), and ADC (ρ = 0.325, *p* < 0.001). D* showed a weaker but significant correlation with EDSS (ρ = 0.253, *p* < 0.001), whereas f was not significantly correlated with EDSS as a single marker (ρ = −0.079, *p* = 0.278). These findings were broadly consistent with the primary ROC-based analysis, supporting the dominant contribution of fractional volumetry and lesion burden, with IVIM-derived f interpreted as exploratory and additive within combined models.

### 3.2. Single-Marker ROC Analysis for Identifying EDSS ≥ 3

Table 2 presents the single-marker ROC analysis for identifying patients with EDSS ≥ 3 using lesion count, volumetric MRI measures, and IVIM-DWI metrics. Lesion count demonstrated an AUC of 0.696, with an optimal cutoff of ≥16 lesions, sensitivity of 49.1%, specificity of 82.4%, and Youden index of 0.314.

Fractional volumetric measures showed the highest single-marker AUC values. BPF and CSF fraction each achieved an AUC of 0.705, with optimal cutoffs of ≤75.90% for BPF and ≥24.10% for CSF fraction. Both markers showed identical sensitivity, specificity, and Youden index values.

Among absolute volumetric measures, CSF volume showed the highest AUC (0.674), followed by BPV (0.629), GM volume (0.622), and WM volume (0.615). BV showed minimal discriminatory performance, with an AUC of 0.503.

Among IVIM-DWI metrics, D and ADC demonstrated AUC values of 0.653 and 0.642, respectively. D* and f showed lower AUC values of 0.589 and 0.576, respectively. Detailed single-marker ROC results are presented in Table 2.

### 3.3. Combined ROC Model Performance for Identifying Patients with EDSS ≥ 3

Table 3 summarizes the performance of selected combined ROC models integrating lesion count, volumetric MRI measures, and IVIM-DWI metrics for identifying patients with EDSS ≥ 3. The lesion count + BPF model achieved an AUC of 0.757 and a 5-fold cross-validated AUC of 0.737, with a sensitivity of 75.5%, specificity of 68.4%, accuracy of 70.4%, and Youden index of 0.439.

When IVIM-DWI parameters were added separately to the lesion count + BPF model, the ADC and D models showed AUC values comparable to the lesion count + BPF model. The lesion count + BPF + D* model achieved an AUC of 0.768 and a 5-fold cross-validated AUC of 0.738. The lesion count + BPF + f model showed the numerically highest cross-validated AUC, with an AUC of 0.769 and a 5-fold cross-validated AUC of 0.746.

At the optimal cutoff, the lesion count + BPF + f model achieved a sensitivity of 75.5%, specificity of 67.6%, accuracy of 69.8%, and Youden index of 0.431. The broader clinical-imaging model incorporating age, sex, DMT status, lesion count, and BPF achieved an AUC of 0.767 and a 5-fold cross-validated AUC of 0.737, with higher specificity and accuracy than the imaging-only models. Detailed model performance is presented in Table 3. To account for potential clinical confounding, an additional adjusted clinical-imaging model including age, sex, disease duration, DMT status, lesion count, BPF, and f was evaluated among patients with available disease-duration data (*n* = 185). As shown in Table 3, this model achieved an AUC of 0.781 and a 5-fold cross-validated AUC of 0.722, with higher specificity and accuracy than the imaging-only lesion count + BPF + f model but lower cross-validated AUC. The adjusted odds ratios, confidence intervals, and *p*-values for the variables included in this model are provided in Supplementary Table S2.

The AUC difference between the lesion count + BPF + f model and the lesion count + BPF model was 0.012. Paired bootstrap analysis showed that this difference was not statistically significant (95% CI: −0.015 to 0.041; *p* = 0.377). The cross-validated AUC difference was also small (0.746 versus 0.737), supporting a cautious interpretation of the incremental contribution of f.

### 3.4. Exploratory Tissue-Specific Volumetric–IVIM Models

Exploratory combined ROC models were additionally evaluated to assess the performance of tissue-specific volumetric measures, including GM volume, WM volume, and BPV, when integrated with ADC or D. These analyses were performed to examine whether absolute tissue-specific volumetric measures combined with lesion-averaged IVIM-DWI metrics improved stratification of patients with EDSS ≥ 3.

Overall, models incorporating GM volume, WM volume, or BPV with ADC or D did not outperform the main multimodal MRI model based on lesion count, BPF, and f. The highest exploratory AUC was observed for lesion count + BPV + D, followed by lesion count + GM volume + ADC and lesion count + GM volume + D. Detailed exploratory model performance is provided in Supplementary Table S3.

## 4. Discussion

This study evaluated lesion burden, volumetric MRI measures, and IVIM-DWI metrics for identifying EDSS ≥ 3 status in RRMS. Fractional volumetric measures outperformed absolute BV as single markers, lesion count provided comparable information, and the highest cross-validated AUC was observed for the lesion count + BPF + f model. Overall, the findings indicate that focal lesion burden and proportional brain tissue loss are the main imaging components associated with EDSS ≥ 3, whereas f provides only modest complementary value. The overall model performance was moderate, and the incremental gain after adding f was not statistically significant and should therefore be considered exploratory.

The stronger performance of fractional volumetric measures compared with absolute BV highlights the importance of proportional indices in cross-sectional disability stratification. BPF and CSF fraction should be interpreted as complementary expressions of the same volumetric relationship rather than independent markers: lower BPF reflects reduced relative brain parenchymal preservation, whereas higher CSF fraction reflects proportional CSF space expansion. Their identical ROC performance is therefore expected and supports the same underlying interpretation of greater relative tissue loss among patients with EDSS ≥ 3. Absolute BV, in contrast, may be influenced by inter-individual variability in head size, premorbid brain reserve, age, and sex, which can reduce its ability to distinguish disability groups at a single time point. These findings are consistent with previous work linking brain atrophy to disability accumulation in MS and with consensus recommendations emphasizing the clinical relevance of brain atrophy measures while acknowledging methodological challenges in their implementation [24,26,27]. In this cohort, the weak discriminatory value of BV further supports the relevance of normalized volumetric measures for EDSS-based disability stratification.

Lesion count also showed meaningful discriminatory performance, consistent with the established role of focal inflammatory lesion burden in MS-related disability. However, lesion count did not outperform fractional volumetric measures, supporting the concept that visible lesion burden alone does not fully explain disability severity in MS. This observation is aligned with the clinico-radiological paradox, in which conventional lesion metrics show an incomplete relationship with clinical impairment because EDSS reflects the cumulative effects of focal lesions, diffuse normal-appearing tissue injury, GM pathology, network disruption, spinal cord involvement, and neurodegenerative tissue loss [15,18]. In this cohort, the higher AUC of the lesion count + BPF model supports the complementary value of focal lesion burden and proportional brain tissue loss. The adjusted clinical-imaging analysis further supported the central role of lesion burden and proportional brain tissue loss. After accounting for age, sex, disease duration, and DMT status, lesion count and BPF remained independently associated with EDSS ≥ 3, whereas f showed a borderline association. These findings reinforce the interpretation that lesion count and BPF represent the most consistent imaging components of the model, while the added contribution of f appears modest after clinical adjustment.

The contribution of IVIM-DWI metrics provides further insight into the potential value of microstructural and perfusion-related lesion information. As single markers, ADC and D showed modest discriminatory performance, whereas D* and f were weaker individually. However, when IVIM-DWI parameters were integrated with lesion count and BPF, perfusion-related metrics showed greater relevance, with the lesion count + BPF + f model demonstrating the highest cross-validated AUC and the lesion count + BPF + D* model showing closely comparable performance. This pattern suggests that IVIM-DWI metrics may be more informative when interpreted alongside lesion count and BPF rather than as isolated markers. Physiologically, f and D* may reflect perfusion-related or microvascular signal components within lesions, which could complement structural measures of lesion burden and brain tissue loss. These findings are consistent with the broader rationale of IVIM-DWI as a technique that separates diffusion-related and perfusion-related signal components [28,29,30,31], and with previous MS studies showing that IVIM-derived metrics can characterize lesion heterogeneity and relate to clinical disability measures [32,33,34]. Nevertheless, the improvement over the lesion count + BPF model was small and was not statistically significant in paired bootstrap analysis. Together with the weak single-marker performance of f and the small cross-validated AUC difference, this supports a cautious interpretation in which IVIM-DWI may provide complementary information, but its incremental value requires confirmation in larger external cohorts.

Exploratory tissue-specific models provided additional context regarding the relative contribution of absolute GM, WM, and BPV measures when combined with IVIM-DWI metrics. Previous studies have shown that GM and WM atrophy are clinically relevant in MS and may be associated with disability accumulation, with GM loss, particularly in deep GM structures, often showing strong links to clinical worsening [41,42]. However, the clinical performance of tissue-specific absolute volumes may vary according to segmentation approach, scanner variability, disease phenotype, and the disability measure used [25,26,27]. In the present study, models incorporating GM, WM, or BPV with ADC or D did not outperform the main multimodal model. This suggests that absolute tissue-specific volumes captured relevant disease-related tissue loss but did not provide superior EDSS ≥ 3 stratification compared with BPF when combined with lesion burden and IVIM-DWI metrics. Therefore, BPF appears to provide a more robust summary of proportional brain tissue preservation in the combined imaging models, while the exploratory GM, WM, and BPV models did not show superior performance in this cohort.

The ROC-based framework used in this study adds a clinically relevant perspective beyond conventional association analyses. A marker may show a statistically significant relationship with EDSS but still demonstrate limited ability to distinguish patients across a clinically meaningful disability threshold. By focusing on EDSS ≥ 3, the present analysis evaluated whether each imaging marker, and each combined model, could distinguish patients across this threshold rather than simply demonstrating correlation with clinical severity. This distinction is important because EDSS is an ordinal and nonlinear scale, and modest changes in imaging measures may not translate into equivalent clinical separation across disability strata [6,7]. The use of a unified ROC framework is therefore a key strength of this study, enabling direct comparison of lesion-based, volumetric, and IVIM-DWI markers within the same cohort. It is important to emphasize that the combined ROC models were intended for cross-sectional disability stratification rather than diagnosis or longitudinal prediction. Accordingly, these models should be interpreted as internally evaluated exploratory models for identifying current EDSS-defined disability status within this cohort, not as prognostic tools or clinically validated decision-making models. Therefore, the reported AUC values should be interpreted as measures of discriminatory performance for identifying patients with EDSS ≥ 3 within this cohort, not as evidence of prognostic utility.

The supplementary EDSS-based correlation analysis provided additional context across the full disability range and showed a pattern broadly consistent with the primary EDSS ≥ 3 ROC-based analysis. The strongest volumetric association was observed for the complementary BPF/CSF fraction relationship, reflecting lower relative brain parenchymal preservation and greater proportional CSF space expansion with increasing EDSS. Lesion count and diffusion-related IVIM-DWI metrics, particularly D and ADC, also showed significant but more modest correlations with EDSS. In contrast, f was not significantly correlated with EDSS as a single marker, reinforcing the interpretation that its contribution is exploratory and additive within combined models rather than dominant as an isolated marker. These supplementary findings support the use of EDSS ≥ 3 as a clinically interpretable ROC threshold while also acknowledging that dichotomization does not fully capture the ordinal disability spectrum.

Several limitations should be acknowledged. First, the retrospective cross-sectional design limits interpretation to EDSS-based disability stratification at a single time point; therefore, longitudinal and multicenter studies are needed to evaluate the stability of these multimodal MRI models over time, particularly because prior longitudinal studies have shown that brain atrophy and lesion burden provide complementary information for long-term disability outcomes in MS [43,44]. In addition, the use of a single 1.5-T MRI scanner may limit generalizability because lesion detection, IVIM-DWI parameter estimation, and volumetric measurements can be influenced by scanner field strength and acquisition quality. Explicit lesion filling was not performed before CAT12 segmentation; although all cases were processed using the same standard CAT12 pipeline with segmentation plausibility checks, this may have affected tissue classification and volumetric estimates, particularly in patients with higher lesion burden. Second, the complete-case design may introduce selection bias, and external validation in independent cohorts is required. Although an adjusted clinical-imaging analysis was added to account for age, sex, disease duration, and DMT status, residual confounding cannot be excluded because of the retrospective cross-sectional design and the incomplete availability of disease-duration data for a small number of patients. Detailed DMT class, treatment duration, and escalation history were not consistently available, and treatment-related residual confounding cannot be excluded. Third, EDSS is an ordinal and ambulation-weighted measure, and dichotomizing EDSS at 3 may reduce information from the full disability range compared with continuous or ordinal EDSS analyses. EDSS-to-MRI timing was determined retrospectively from routine clinical documentation rather than through a prospectively standardized same-day research assessment, which may have introduced temporal variability between clinical disability scoring and MRI-derived measures. In addition, the EDSS ≥ 3 group was smaller than the EDSS < 3 group, which may have limited the stability of multivariable model estimates despite the use of parsimonious models and internal cross-validation. Although 5-fold cross-validation was used to estimate internal model stability, this approach does not replace repeated cross-validation, nested validation strategies, or external validation in independent cohorts. Because multiple group comparisons and ROC analyses were performed, the possibility of false-positive findings cannot be excluded, and the results should be interpreted as exploratory. Finally, lesion count was used as the available marker of focal lesion burden and did not account for lesion volume or size, lesion location, infratentorial involvement, cortical lesions, or spinal cord lesions. Lesion-volume analysis was not performed because a dedicated 3D FLAIR-based lesion-volumetry workflow was not available in this retrospective dataset. Lesion-averaged IVIM-DWI metrics may also not fully capture lesion subtype or regional heterogeneity. Normal-appearing white matter, cortical GM, and deep GM IVIM-DWI values were not assessed because the IVIM-DWI analysis was lesion-based. Formal inter-rater or intra-rater ROI reproducibility analysis was not performed. The absence of spinal cord imaging, cognitive assessment, and regional deep GM volumetry may also have limited disability-related characterization, particularly because thalamic atrophy has been associated with subsequent disability progression in MS [45].

Future studies should validate these findings in larger, independent, and longitudinal cohorts incorporating additional disability-relevant clinical and imaging measures, such as spinal cord MRI, cognitive assessment, and regional deep GM volumetry.

## 5. Conclusions

Fractional volumetric measures, particularly BPF and CSF fraction, showed stronger discriminatory performance than absolute brain volume measures for EDSS ≥ 3 stratification in RRMS. The lesion count + BPF + f model achieved the highest cross-validated performance among the evaluated MRI models, although its incremental improvement over lesion count + BPF was modest and not statistically significant. IVIM-DWI metrics provided complementary but non-dominant discriminatory information, with ADC and D showing modest single-marker performance and f contributing mainly within combined models. These findings support further evaluation of MRI-based cross-sectional disability stratification in larger longitudinal cohorts with external validation.

## Figures and Tables

**Table 1:** Clinical, IVIM-DWI, and volumetric MRI characteristics according to EDSS group.

Variable	EDSS < 3 (*n* = 136)	EDSS ≥ 3 (*n* = 53)	*p*-Value	Cohen’s d
Age, years	35.13 ± 8.92	38.30 ± 9.98	0.047	0.34
Sex, female, *n* (%)	97 (71.3)	35 (66.0)	0.477	—
DMT, treated, *n* (%)	98 (72.1)	40 (75.5)	0.635	—
Disease duration, years	5.65 ± 5.26	7.91 ± 4.60	0.005	0.44
Number of MS lesions	9.87 ± 6.86	15.93 ± 9.67	<0.001	0.78
ADC, ×10^−3^ mm^2^/s	1.08 ± 0.13	1.15 ± 0.17	0.004	0.53
D, ×10^−3^ mm^2^/s	1.02 ± 0.13	1.10 ± 0.16	0.002	0.57
D*, ×10^−3^ mm^2^/s	1.25 ± 0.15	1.30 ± 0.19	0.084	0.31
f, %	6.00 ± 1.80	5.50 ± 1.20	0.015	−0.33
BV, mL	1400.31 ± 138.58	1392.17 ± 141.27	0.721	−0.06
GM volume, mL	611.26 ± 68.04	578.43 ± 71.33	0.005	−0.48
WM volume, mL	495.51 ± 66.56	469.95 ± 75.85	0.034	−0.37
BPV, mL	1106.77 ± 122.45	1048.38 ± 133.23	0.007	−0.47
CSF volume, mL	293.53 ± 67.92	343.79 ± 84.04	<0.001	0.69
CSF fraction, %	20.90 ± 4.30	24.70 ± 5.30	<0.001	0.81
BPF, %	79.10 ± 4.30	75.30 ± 5.30	<0.001	−0.81

Note: Values are presented as mean ± standard deviation or *n* (%) as appropriate. Cohen’s d was reported only for continuous variables. ADC = apparent diffusion coefficient; D = true diffusion coefficient; D* = pseudo-diffusion coefficient; f = perfusion fraction; BV = brain volume; GM = gray matter; WM = white matter; BPV = brain parenchymal volume; CSF = cerebrospinal fluid; BPF = brain parenchymal fraction; DMT = disease-modifying therapy.

**Table 2:** Single-marker ROC analysis for identifying patients with EDSS ≥ 3.

Marker Group	Marker	AUC	95% CI	Optimal Cutoff for EDSS ≥ 3	Sensitivity	Specificity	Youden Index	*p*-Value
Lesion burden	Lesion count	0.696	0.608–0.784	≥16.00	0.491	0.824	0.314	<0.001
Fractional volumetric measures	CSF fraction	0.705	0.618–0.792	≥24.10	0.547	0.772	0.319	<0.001
	BPF	0.705	0.618–0.792	≤75.90	0.547	0.772	0.319	<0.001
Absolute volumetric measures	CSF volume	0.674	0.585–0.764	≥332.18	0.547	0.772	0.319	<0.001
	BPV	0.629	0.538–0.721	≤1046.67	0.604	0.640	0.243	0.006
	GM volume	0.622	0.530–0.713	≤559.30	0.415	0.787	0.202	0.009
	WM volume	0.615	0.523–0.707	≤404.40	0.264	0.941	0.205	0.014
	BV	0.503	0.411–0.595	≥1364.63	0.623	0.471	0.093	0.954
IVIM metrics	D	0.653	0.563–0.744	≥1.03	0.736	0.566	0.302	0.001
	ADC	0.642	0.552–0.733	≥1.20	0.434	0.838	0.272	0.002
	D*	0.589	0.497–0.681	≥1.38	0.434	0.838	0.272	0.058
	f	0.576	0.483–0.669	≤5.70	0.604	0.566	0.170	0.105

Note: ADC, D, and D* are reported in 10^−3^ mm^2^/s. BV, GM volume, WM volume, BPV, and CSF volume are reported in mL. CSF fraction, BPF, and f are reported as percentages. The *p*-value indicates whether the AUC differed significantly from 0.5. Cutoff values are exploratory Youden-derived thresholds and should not be interpreted as validated or clinically actionable decision thresholds. AUC = area under the curve; CI = confidence interval; EDSS = Expanded Disability Status Scale; ADC = apparent diffusion coefficient; D = true diffusion coefficient; D* = pseudo-diffusion coefficient; BV = brain volume; GM = gray matter; WM = white matter; BPV = brain parenchymal volume; CSF = cerebrospinal fluid; BPF = brain parenchymal fraction; f = perfusion fraction.

**Table 3:** Performance of selected combined ROC models integrating lesion burden, volumetric MRI, and IVIM-DWI metrics for identifying patients with EDSS ≥ 3.

Model	AUC	95% CI	5-Fold CV AUC	Sensitivity	Specificity	Accuracy	Youden Index
Lesion count + BPF	0.757	0.677–0.836	0.737	0.755	0.684	0.704	0.439
Lesion count + CSF volume	0.743	0.663–0.821	0.723	0.679	0.735	0.720	0.415
Lesion count + BPF + ADC	0.758	0.678–0.832	0.733	0.717	0.713	0.714	0.430
Lesion count + BPF + D	0.757	0.677–0.835	0.731	0.774	0.669	0.698	0.443
Lesion count + BPF + D*	0.768	0.693–0.840	0.738	0.717	0.713	0.714	0.430
Lesion count + BPF + f	0.769	0.692–0.844	0.746	0.755	0.676	0.698	0.431
Age + sex + DMT status + lesion count + BPF	0.767	0.684–0.845	0.737	0.679	0.816	0.778	0.495
Age + sex + disease duration + DMT status + lesion count + BPF + f	0.781	0.699–0.852	0.722	0.725	0.784	0.768	0.509

Note: AUC values represent model-level discriminatory performance, whereas 5-fold CV AUC values represent internally cross-validated performance. Sensitivity, specificity, accuracy, and Youden index were calculated at the optimal exploratory model cutoff. The lesion count + BPF model was evaluated as the core imaging model combining focal lesion burden and fractional brain tissue loss. ADC, D, D*, and f were added separately to this framework to assess the contribution of individual IVIM-DWI parameters. The adjusted clinical-imaging model including disease duration was evaluated among patients with available disease-duration data (*n* = 185; EDSS < 3, *n* = 134; EDSS ≥ 3, *n* = 51). This adjusted model focused on f because lesion count + BPF + f showed the highest cross-validated performance among the IVIM-augmented imaging models; other IVIM-DWI parameters were not entered simultaneously to avoid overfitting and potential collinearity. DMT status was treated as a binary variable indicating treated versus untreated status. Multivariable ROC models are exploratory internally evaluated stratification models and should not be interpreted as clinically actionable, diagnostic, prognostic, or treatment decision-making models. AUC = area under the curve; CI = confidence interval; CV = cross-validated; EDSS = Expanded Disability Status Scale; BPF = brain parenchymal fraction; CSF = cerebrospinal fluid; ADC = apparent diffusion coefficient; D = true diffusion coefficient; D* = pseudo-diffusion coefficient; f = perfusion fraction; DMT = disease-modifying therapy.

## Data Availability

The datasets generated and/or analyzed during the current study are available from the corresponding author upon reasonable request, subject to institutional and ethical restrictions.

## Supplementary Materials

The following supporting information can be downloaded at: https://www.mdpi.com/article/10.3390/brainsci16070743/s1, Table S1: Full EDSS distribution and supplementary Spearman correlations with EDSS; Table S2: Adjusted clinical-imaging logistic regression model for identifying patients with EDSS ≥ 3; Table S3: Exploratory tissue-specific volumetric–IVIM ROC models for identifying patients with EDSS ≥ 3.

## Abbreviations

The following abbreviations are used in this manuscript:

MRI	Magnetic Resonance Imaging
IVIM-DWI	Intravoxel incoherent motion diffusion-weighted imaging
RRMS	Relapsing–remitting multiple sclerosis
EDSS	Expanded Disability Status Scale
ROC	Receiver Operating Characteristic
AUC	Area Under Curve
ADC	Apparent Diffusion Coefficient
D	True diffusion coefficient
D*	Pseudo-diffusion coefficient
f	Perfusion fraction
MS	Multiple Sclerosis
GM	Gray matter
WM	White Matter
CSF	Cerebrospinal fluid
BPF	Brain parenchymal fraction
