# Supplementary material for: MRI Markers of EDSS ≥ 3 in Relapsing–Remitting Multiple Sclerosis: An Assessment of Lesion Burden, Brain Volumetry, and IVIM-DWI Metrics
Source: Brain Sci. 2026 Jul 14;16(7):743. doi: 10.3390/brainsci16070743 (PMC13406835; doi:10.3390/brainsci16070743)
Supplement: Supplementary file 1 [file brainsci-16-00743-s001.zip › brainsci-4405017-supplementary.pdf]

Supplementary Table S1. Full EDSS distribution and supplementary Spearman correlations with EDSS.

A. Full EDSS distribution in the complete-case cohort (n = 189).

EDSS score	n
0	34
1	33
1.5	9
2	42
2.5	18
3	18
3.5	7
4	2
4.5	3
5	1
5.5	3
6	6
6.5	4
7	6
8	3

B. Supplementary Spearman correlations between EDSS and the main MRI markers.

MRI marker	Spearman's ϱ	p-value
Lesion count	0.368	<0.001
CSF fraction	0.411	<0.001
BPF	−0.410	<0.001
ADC	0.325	<0.001
D	0.339	<0.001
D*	0.253	<0.001
f	−0.079	0.278

Note: EDSS was treated as an ordinal/continuous disability measure in this supplementary sensitivity analysis. ADC, D, and D* are IVIM-DWI parameters; BPF = brain parenchymal fraction; CSF = cerebrospinal fluid; f = perfusion fraction.

Supplementary Table S2. Adjusted clinical-imaging logistic regression model for identifying patients with EDSS ≥3.

Variable included in the adjusted model	OR	95% CI	p-value
Age, per year	1.015	0.971–1.062	0.500
Female sex	0.680	0.311–1.487	0.334
Disease duration, per year	1.020	0.944–1.101	0.622
DMT treated	0.863	0.351–2.121	0.748
Lesion count, per lesion	1.074	1.025–1.125	0.003
BPF, per 1% increase	0.890	0.817–0.969	0.007
f, per 1% increase	0.744	0.547–1.013	0.060

Note: The model included patients with available disease-duration data (n = 185; EDSS <3, n = 134; EDSS ≥3, n = 51). Model-level discriminatory performance is reported in Table 3. BPF and f were entered as percentages. DMT status was treated as a binary variable indicating treated versus untreated status. OR = odds ratio; CI = confidence interval; EDSS = Expanded Disability Status Scale; DMT = disease-modifying therapy; BPF = brain parenchymal fraction; f = perfusion fraction.

Supplementary Table S3. Exploratory tissue-specific volumetric–IVIM ROC models for identifying patients with EDSS ≥3.

Model	AUC	95% CI	5-fold CV AUC	Sensitivity	Specificity	Accuracy	Youden index
Lesion count + GM volume + ADC	0.728	0.640–0.817	0.693	0.736	0.721	0.725	0.456
Lesion count + GM volume + D	0.727	0.638–0.815	0.695	0.717	0.728	0.725	0.445
Lesion count + WM volume + ADC	0.723	0.625–0.813	0.689	0.736	0.706	0.714	0.442
Lesion count + WM volume + D	0.724	0.628–0.814	0.687	0.717	0.713	0.714	0.430
Lesion count + BPV + ADC	0.727	0.631–0.815	0.692	0.717	0.735	0.730	0.452
Lesion count + BPV + D	0.729	0.636–0.817	0.692	0.623	0.816	0.762	0.439
GM volume + ADC	0.669	0.576–0.756	0.643	0.604	0.735	0.698	0.339
GM volume + D	0.674	0.580–0.761	0.642	0.604	0.728	0.693	0.332
WM volume + ADC	0.655	0.558–0.753	0.628	0.396	0.919	0.772	0.315
WM volume + D	0.658	0.564–0.754	0.631	0.415	0.897	0.762	0.312
BPV + ADC	0.664	0.568–0.755	0.641	0.642	0.662	0.656	0.303
BPV + D	0.667	0.569–0.756	0.643	0.642	0.676	0.667	0.318

Note: AUC = area under the curve; CI = confidence interval; CV = cross-validated; EDSS = Expanded Disability Status Scale; GM = gray matter; WM = white matter; BPV = brain parenchymal volume; ADC = apparent diffusion coefficient; D = true diffusion coefficient; IVIM-DWI = intravoxel incoherent motion diffusion-weighted imaging.
